# Women with a history of gestational diabetes mellitus have preserved sensory nerve‐mediated dilatation in the cutaneous microvasculature

**DOI:** 10.1113/EP093827

**Published:** 2026-09-23

**Authors:** Grace S. Maurer, Kelsey S. Schwartz, Diana I. Jalal, Anna E. Stanhewicz

**Affiliations:** ^1^ Department of Health, Sport, and Human Physiology University of Iowa Iowa City Iowa USA; ^2^ Carver College of Medicine University of Iowa Iowa City Iowa USA; ^3^ The Iowa City Veteran Affairs Health Care System Iowa City Iowa USA

**Keywords:** gestational diabetes, microvasculature, sensory nerve

## Abstract

Women with a history of gestational diabetes mellitus (GDM) have a greater risk of developing type 2 diabetes mellitus and cardiovascular disease than healthy control (HC) women who had an uncomplicated pregnancy. Microvascular endothelial dysfunction, mediated by reduced nitric oxide (NO)‐dependent mechanisms, persists after GDM. However, it is unknown whether cutaneous sensory nerve‐mediated dilatation is similarly reduced in these women. We hypothesised that women with a history of GDM would exhibit attenuated sensory nerve‐mediated vasodilatation compared with HC women. One intradermal microdialysis fibre was placed in the ventral forearm of 30 HC and 22 GDM women for the local delivery of lactated Ringer's solution during a local heating protocol (42°C; 0.1°C s^−1^). The heater remained at 42°C until blood flow plateaued (∼40 min). Next, the site was perfused with 15 mM *N* ^G^‐nitro‐l‐arginine methyl ester (a NO synthase inhibitor; ∼40 min) to determine NO‐dependent dilatation. Red blood cell flux was measured by laser‐Doppler flowmetry (LDF). Cutaneous vascular conductance was calculated (CVC = LDF/mean arterial pressure) and normalized to maximum (28 mM sodium nitroprusside + local heat 43°C). The initial sensory nerve‐mediated peak response was not different between groups (*P *= 0.623). The endothelium‐dependent plateau (*P* < 0.001) and NO contribution to the plateau (*P* < 0.01) were attenuated in GDM compared with HC women. Our findings suggest that despite reductions in endothelium‐ and NO‐dependent dilatation, women who had GDM do not exhibit impairments in local cutaneous sensory nerve‐mediated vasodilatation compared with HC women.

## Abstract (highlights)

- **What is the central question of this study?**
  Is sensory nerve‐mediated vasodilatation attenuated in the cutaneous microvasculature of women who have had gestational diabetes?
- **What is the main finding and its importance?**
  Women with a history of gestational diabetes exhibit preserved sensory nerve‐mediated vasodilatation in comparison to women with a history of uncomplicated pregnancy. Although women who have had gestational diabetes have reduced microvascular endothelium‐dependent dilatation and are at an increased risk of cardiovascular disease and type 2 diabetes, our findings suggest that the neurogenic response in the cutaneous microvasculature is unaltered in otherwise healthy women within 5 years of pregnancy complicated by gestational diabetes.

## 1 INTRODUCTION

Gestational diabetes mellitus (GDM) is defined as new‐onset glucose intolerance that arises from increased insulin resistance in women during pregnancy (ACOG Practice Bulletin No. 190: Gestational Diabetes Mellitus, 2018). GDM complicates ∼10% of pregnancies in the USA, with incidence rates steadily increasing over the past 5 years (Gregory & Ely, 2022). Although GDM usually resolves within 6 weeks postpartum, 20%–60% of women with prior GDM subsequently develop type 2 diabetes mellitus (T2DM) within 5 years of delivery (Kim et al., 2002). Women with a history of GDM also have an increased likelihood of developing cardiovascular disease (CVD) within the first decade postpartum and exhibit a higher risk of CVD‐related mortality in comparison to healthy control (HC) women who experienced uncomplicated pregnancies (Kramer et al., 2019; Retnakaran & Shah, 2017). Although the association between GDM and future T2DM and CVD risk is clear, the underlying mechanisms driving this accelerated disease progression are poorly understood.

Microvascular endothelial dysfunction is an early predictor of future metabolic (Muris et al., 2012) and macrovascular (Landmesser et al., 2004) dysfunction and is likely to contribute to the increased risk of T2DM and CVD in women with a history of GDM. Overt T2DM is associated with both microvascular endothelial dysfunction (Faselis et al., 2020; Nacci et al., 2009) and sensory nerve dysfunction (Fernyhough & McGavock, 2014; Karki et al., 2016). Importantly, diabetic peripheral neuropathy, defined as dysfunction of the peripheral nervous system in diabetes, is considered a microvascular complication of diabetes (Sempere‐Bigorra et al., 2021), and T2DM‐related sensory neuropathy is associated with microvascular endothelial dysfunction (Cameron et al., 2001; Chan et al., 2006; Faselis et al., 2020; Leung et al., 2016). Chronic hyperglycaemia in T2DM induces neuropathic changes that impair sensory nerve integrity and signalling through numerous different mechanisms, including advanced glycation end‐products, cytokines and oxidative stress (Yagihashi et al., 2011). Furthermore, sensory nerve‐mediated microvascular dilatation can be attenuated in conditions associated with reduced nitric oxide (NO) bioavailability, such as T2DM and hypertension (Hébert et al., 2017; Watson et al., 2002; Wong et al., 2020). Although chronic hyperglycaemia associated with T2DM is a known cause of sensory neuropathy, it is unclear whether the temporary hyperglycaemia (12–16 weeks) of GDM impairs sensory nerve function or confers increased risk of neuropathy in women with a history of GDM, independent of T2DM. However, evidence suggests that peripheral neuropathy begins in the early stages of diabetes pathogenesis, particularly in individuals at high risk for T2DM (Lee et al., 2015), and restoration of glycaemic control fails to reverse peripheral neuropathy in diabetic patients (Ang et al., 2014; Yang et al., 2025).

The human cutaneous circulation is an easily accessible and representative vascular bed suitable for assessing in vivo microvascular function (Holowatz et al., 2008). In humans, non‐painful local heating of the skin is a common, minimally invasive approach to evaluate cutaneous microvascular function. The cutaneous local heating response is characterized by two distinct phases: an initial sensory nerve‐mediated peak, followed by a prolonged secondary endothelium‐dependent plateau (Carter & Hodges, 2011; Hodges et al., 2015). The initial sensory nerve‐mediated response is mediated primarily by direct action of local sensory nerves at transient receptor potential vanilloid (TRPV)‐1 channels, leading to a release of local vasodilators (Houghton et al., 2006; Minson et al., 2001). Downstream of TRPV‐1 receptors, the local axon reflex is also mediated by endothelium‐derived hyperpolarizing factors (Brunt & Minson, 2012) and is 20% NO dependent. In contrast, the secondary endothelium‐dependent plateau response to local heating is 60%–80% dependent on NO. Work from our laboratory has previously shown that women with a history of GDM exhibit a reduced cutaneous microvascular endothelium‐dependent dilatation (secondary plateau) response to local heating, mediated by reductions in NO‐dependent dilatation (Stanhewicz et al., 2022). However, to date, no studies have examined whether the initial sensory nerve‐mediated peak response to local heating is altered in women with a history of GDM.

The primary purpose of this study was to assess whether local sensory nerve‐mediated vasodilatation during non‐painful local heating of the skin is impaired in otherwise healthy women with a history of GDM. We hypothesised that in response to local heating, otherwise healthy women with a history of GDM would have an attenuated initial sensory nerve‐mediated peak response compared with HC women. We hypothesised further that women with a history of GDM would have attenuated endothelium‐ and NO‐dependent dilatation compared with HC women.

## 2 MATERIALS AND METHODS

### 2.1 Ethical approval

We compiled data from participants who took part in registered clinical trials [IRB# 202009357 (NCT05946785), IRB# 202303202 (NCT05937841) and IRB# 202405720 (NCT06547619)] and an additional study in our laboratory (IRB# 202204545) that were all approved by the University of Iowa Institutional Review Board. Investigational New Drug Applications were obtained from the US Food and Drug Administration (IND no. 153,527 and 124,294) for the use of pharmacological agents with intradermal microdialysis. Written and verbal consent were obtained voluntarily from all participants prior to enrolment according to the *Declaration of Helsinki* and the US Code of Federal Regulations, Part 46.

### 2.2 Study population

This was an analysis of data collected in studies that were designed a priori to examine the endothelium‐dependent plateau in the cutaneous vascular conductance (CVC) response to local heating (Schwartz et al., 2025; Stanhewicz et al., 2022). We included 52 healthy women who had delivered within the past 5 years. Twenty‐two of these women had a history of GDM during their most recent pregnancy, diagnosed by their obstetrician in accordance with criteria set by the American College of Obstetrics and Gynecologists (ACOG Practice Bulletin No. 190: Gestational Diabetes Mellitus, 2018). The other 30 had a history of an uncomplicated pregnancy, confirmed by medical chart review, and served as control subjects (HC). Data from 20 HC participants and 7 GDM participants were part of studies that have been published elsewhere (Schwartz et al., 2025; Stanhewicz et al., 2022). Data from the remaining 10 HC participants and 15 GDM participants were from pilot studies and other protocols that have not been published. The sensory nerve‐mediated vasodilatation responses supporting this manuscript have not been assessed or published previously.

Participants underwent a complete medical screening that included a physical examination, lipid profile and blood chemistry. All participants were free of CVD, did not use nicotine‐containing products, were non‐diabetic (HbA1c < 5.7%; fasting glucose ≤ 100 mg dL^−1^) and were not taking medications with primary or secondary cardiovascular effects. Exclusion criteria included a history of pre‐eclampsia, history of gestational hypertension and history of hypertension or diabetes before pregnancy. Race and ethnicity were self‐reported. The homeostatic model assessment of insulin resistance [HOMA‐IR = fasting plasma glucose (in mg dL^−1^) × fasting plasma insulin (in µU mL^−1^)/22.5] was used to assess insulin sensitivity (Gutch et al., 2015).

### 2.3 Experimental procedures

Each subject participated in one laboratory visit. One intradermal microdialysis fibre (CMA31 linear microdialysis probe; Harvard Apparatus, Holliston, MA, USA) was placed into the dermal layer of the ventral forearm for the local delivery of lactated Ringer's solution, followed by 15 mM *N* ^G^‐nitro‐l‐arginine methyl ester (l‐NAME; Tocris Bioscience, Minneapolis, MN, USA) for the inhibition of NO synthase. Pharmacological agents were mixed immediately before use, dissolved in lactated Ringer's solution, sterilized using syringe microfilters (Acrodisc; Pall, Ann Arbor, MI, USA), wrapped in foil to prevent photodegradation, and perfused through the microdialysis fibre at a rate of 2 µL min^−1^ (Bee Hive controller and Baby Bee microinfusion pumps; Bioanalytical Systems, West Lafayette, IN, USA). The experimental protocol commenced after an initial hyperaemia‐resolution period (∼60 min). Cutaneous red blood cell flux was measured continually, directly over the microdialysis site, with an integrated laser‐Doppler flowmetry (LDF) probe placed in a local heating unit (Moor Instruments, Wilmington, DE, USA) for the control of local skin temperature. Automated brachial blood pressure (Cardiocap; GE Healthcare, Milwaukee, WI, USA) was measured every 5 min on the contralateral arm throughout the protocol.

### 2.4 Local heating protocol

**FIGURE 1:**
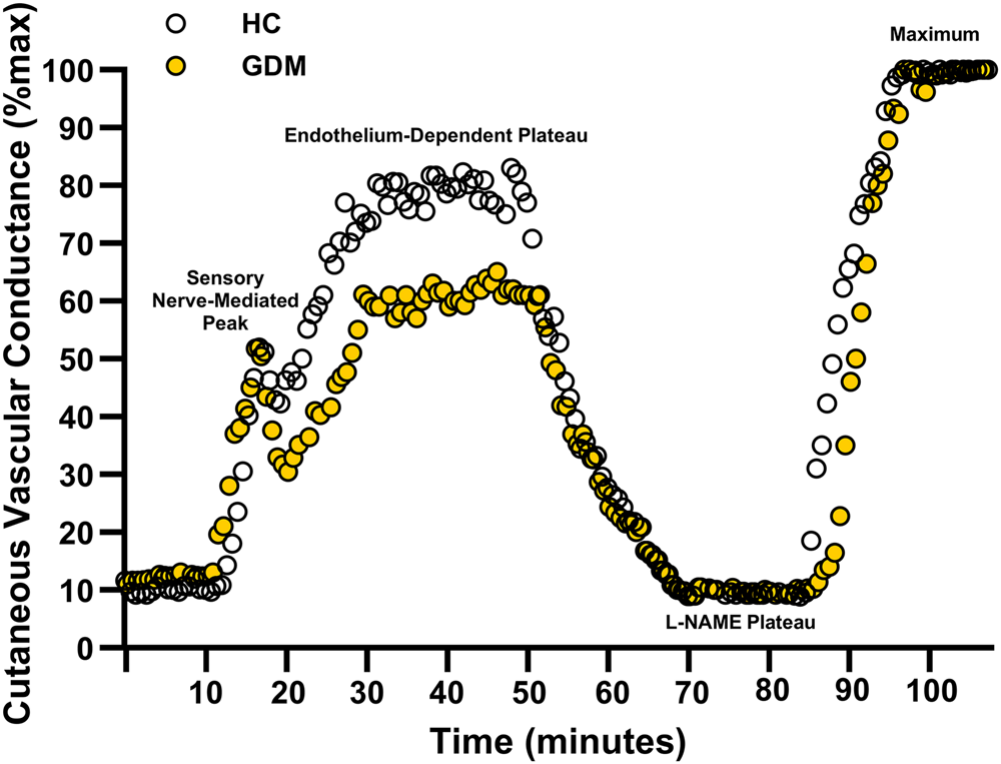
Individual recordings showing representative initial sensory nerve‐mediated peak and sustained endothelium‐dependent plateau responses in the cutaneous microvasculature during standardized local heating of the skin to 42°C. Abbreviations: HC, history of uncomplicated pregnancy; GDM, history of gestational diabetes mellitus; l‐NAME, *N* ^G^‐nitro‐l‐arginine methyl ester.

During baseline measurements, the local heater was set to thermoneutral (33°C). Following stable baseline measurements (∼10 min), the temperature of the local heater was raised to 42°C (0.5°C s^−1^) following a standardized skin local heating protocol (Kellogg et al., 2008; Stanhewicz et al., 2017b) and remained at 42°C until skin blood flow reached a stable plateau (∼40 min, endothelium‐dependent plateau). Next, the microdialysis fibre was perfused with 15 mM l‐NAME until skin blood flow reached a stable plateau (∼40 min, l‐NAME plateau) to determine NO‐dependent dilatation. At the end of the experimental protocol, 28 mmol L^−1^ sodium nitroprusside (United States Pharmacopeia) was perfused, and local temperature was increased to 43°C to elicit maximal dilatation (Stanhewicz et al., 2017a, 2017b). Figure 1 depicts representative recordings of the skin blood flow response to local heating in HC and GDM.

### 2.5 Statistical analysis

All data collection and analysis procedures were standardized before testing, and personnel were blinded to groups during analysis. Blood flow data were digitized at 40 Hz, recorded, and stored for offline analysis (PowerLab and LabChart, ADInstruments, Sydney, NSW, Australia). CVC was calculated (laser‐Doppler flux/mean arterial pressure) and normalized to a percentage of maximum (%max). NO‐dependent dilatation was calculated as the difference between the endothelium‐dependent plateau and the l‐NAME plateau (∆CVC, %max). Student's unpaired *t*‐tests were used to compare responses between groups (SPSS Statistics v.26; IBM Corp., Armonk, NY, USA). Data are presented as means ± SD.

To examine the potential role for body mass index (BMI), fasting plasma glucose and HbA1c to explain our findings in GDM, we ran an ANCOVA with these variables included as covariates. Only BMI was significant for the sensory nerve‐mediated peak (CVC %max, *P *= 0.00382) and the endothelium‐dependent plateau (CVC %max, *P *= 0.00264), and the ANCOVA results did not change the interpretation of the data.

### 2.6 Sample size and power

Because this was a secondary analysis of previously collected cutaneous vascular responses to local heat, no a priori sample size calculation was conducted. We therefore conducted a *post hoc* power analysis to determine whether we were adequately powered to detect differences between HC and GDM where they exist. Because previous studies have demonstrated reduced endothelium‐ and NO‐mediated microvascular vasodilatation in women with a history of GDM (Stanhewicz et al., 2022), we used the local heating plateau data from the present study (Cohen's *d* = 1.09) to determine that our sample yields a power of 1 − *b* = 0.97.

## 3 RESULTS

Participant characteristics are presented in Table 1. There were no differences in age (*P *= 0.118), parity (*P *= 0.183), time postpartum (*P *= 0.832), blood pressure (*P *= 0.511), high‐density lipoprotein (*P *= 0.563), low‐density lipoprotein (*P *= 0.814), fasting triglycerides (*P *= 0.113) or fasting plasma insulin (*P *= 0.0675) between groups. Fasting insulin and HOMA‐IR were measured in a subset of participants (HC, *n* = 19 participants; GDM, *n* = 21 participants). BMI (*P *= 0.0109), fasting plasma glucose (*P *= 0.0210), HOMA‐IR (*P *= 0.0371) and glycated haemoglobin (HbA1c, *P *= 0.0200) were higher in the GDM group compared with the HC group. However, all blood chemistry values were within normal clinical ranges. Nineteen HC participants (12 intrauterine device, 6 oral pill and 1 implant) and nine GDM participants (eight intrauterine device and one implant) were using contraceptives at the time of testing. Six HC and five GDM participants were actively breastfeeding at study enrollment.

**TABLE 1:** Participant characteristics.

Characteristic	HC (*n* = 30 participants)	GDM (*n* = 22 participants)	*P*‐value
Age, years	35 ± 3	33 ± 4	0.118
Parity, number	2 ± 1	2 ± 1	0.183
Race, *n* (%)			
White	28 (94)	21 (95)	
Black or African American	0 (0)	0 (0)	
Asian	1 (3)	1 (5)	
Unknown/not reported	1 (3)	0 (0)	
Ethnicity, *n* (%)			
Not Hispanic or Latino	29 (97)	20 (90)	
Hispanic or Latino	1 (3)	2 (10)	
Time postpartum, months	24 ± 15	24 ± 14	0.832
Glucose management during pregnancy, *n*			
Diet	–	17	
Insulin injection	–	4	
Metformin	–	1	
MAP, mmHg	83 ± 11	85 ± 8	0.511
SBP, mmHg	112 ± 12	115 ± 13	0.342
DBP, mmHg	70 ± 11	73 ± 6	0.345
BMI, kg m^−2^	27.7 ± 5.1	32.2 ± 7.2	**0.0109**
Total cholesterol, mg dL^−1^	175 ± 27	178 ± 34	0.716
HDL, mg dL^−1^	57 ± 12	55 ± 14	0.563
LDL, mg dL^−1^	102 ± 27	104 ± 28	0.814
Triglycerides, mg dL^−1^	76 ± 36	94 ± 43	0.113
Fasting glucose, mg dL^−1^	81 ± 8	86 ± 7	**0.0210**
Fasting insulin, µU mL^−1^	8 ± 6	12 ± 8	0.0675
HOMA‐IR	1.6 ± 1.1	2.6 ± 1.7	**0.0371**
HbA1c, %	5.1 ± 0.2	5.3 ± 0.4	**0.0200**
Contraceptive use, *n*			
Oral pill	6	0	
Implant	1	1	
Intrauterine device	12	8	

*Notes*: Values are the mean ± SD. Fasting insulin and HOMA‐IR were measured in a subset of participants (HC, *n* = 19/30 participants; GDM, *n* = 21/22 participants). Statistical comparisons and *P*‐values are presented from Student's *t* test. The *P*‐values indicated in bold are considered significant.

Abbreviations: BMI, body mass index; DBP, diastolic blood pressure; GDM, history of gestational diabetes mellitus; Hba1c, glycated haemoglobin; HC, history of healthy pregnancy; HDL, high‐density lipoprotein; HOMA‐IR, homeostatic model assessment of insulin resistance; LDL, low‐density lipoprotein; MAP, mean arterial pressure; SBP, systolic blood pressure.

Absolute CVC values at all phases of the local heating protocol are presented in Table 2. There were no differences between the HC or GDM groups in baseline (*P *= 0.578), sensory nerve‐mediated peak (*P *= 0.0565), endothelium‐dependent plateau (*P *= 0.160) or l‐NAME plateau (*P *= 0.137) CVC responses. Maximal CVC was higher in the GDM group compared with the HC group (GDM, 2.3 ± 1.0 CVC vs. HC, 1.8 ± 0.8 CVC; *P *= 0.00649).

**TABLE 2:** Absolute cutaneous vascular conductance at all phases of local heating.

Time point	HC (*n* = 30 participants)	GDM (*n* = 22 participants)	*P*‐value
Baseline	0.2 ± 0.1	0.3 ± 0.2	0.258
Sensory nerve‐mediated peak	1.0 ± 0.5	1.3 ± 0.7	0.0565
Endothelium‐dependent plateau	1.5 ± 0.7	1.8 ± 0.8	0.160
l‐NAME plateau	0.3 ± 0.2	0.4 ± 0.4	0.137
Maximum	1.8 ± 0.8	2.3 ± 1.0	0.00649^*^

*Notes*: Values are the mean ± SD. Cutaneous vascular conductance (flux mmHg^−1^) = laser‐Doppler flux/mean arterial pressure.

Abbreviations: GDM, history of gestational diabetes; HC, history of uncomplicated pregnancy; l‐NAME, *N* ^G^‐nitro‐l‐arginine methyl ester.

* *P *< 0.05 HC vs. GDM.

**FIGURE 2:**
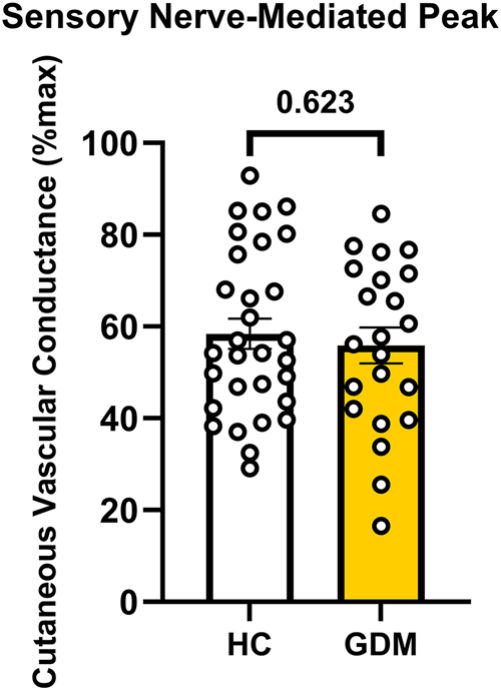
Mean ± SEM of the initial sensory nerve‐mediated vasodilatory peak (cutaneous vascular conductance, % of maximum) induced by standard local heating in the cutaneous microvasculature (42°C) in women with a history of gestational diabetes mellitus (GDM, *n* = 22 participants) and control women who had an uncomplicated pregnancy (HC, *n* = 30 participants) *P *= 0.623.

Figure 2 presents the initial sensory nerve‐mediated peak of the local heating response (CVC, %max). There was no difference in the initial sensory nerve‐mediated peak between groups (CVC, %max: HC, 58.4 ± 18.0 vs. GDM, 55.9 ± 18.3; *P *= 0.623, ANCOVA *P *= 0.108).

**FIGURE 3:**
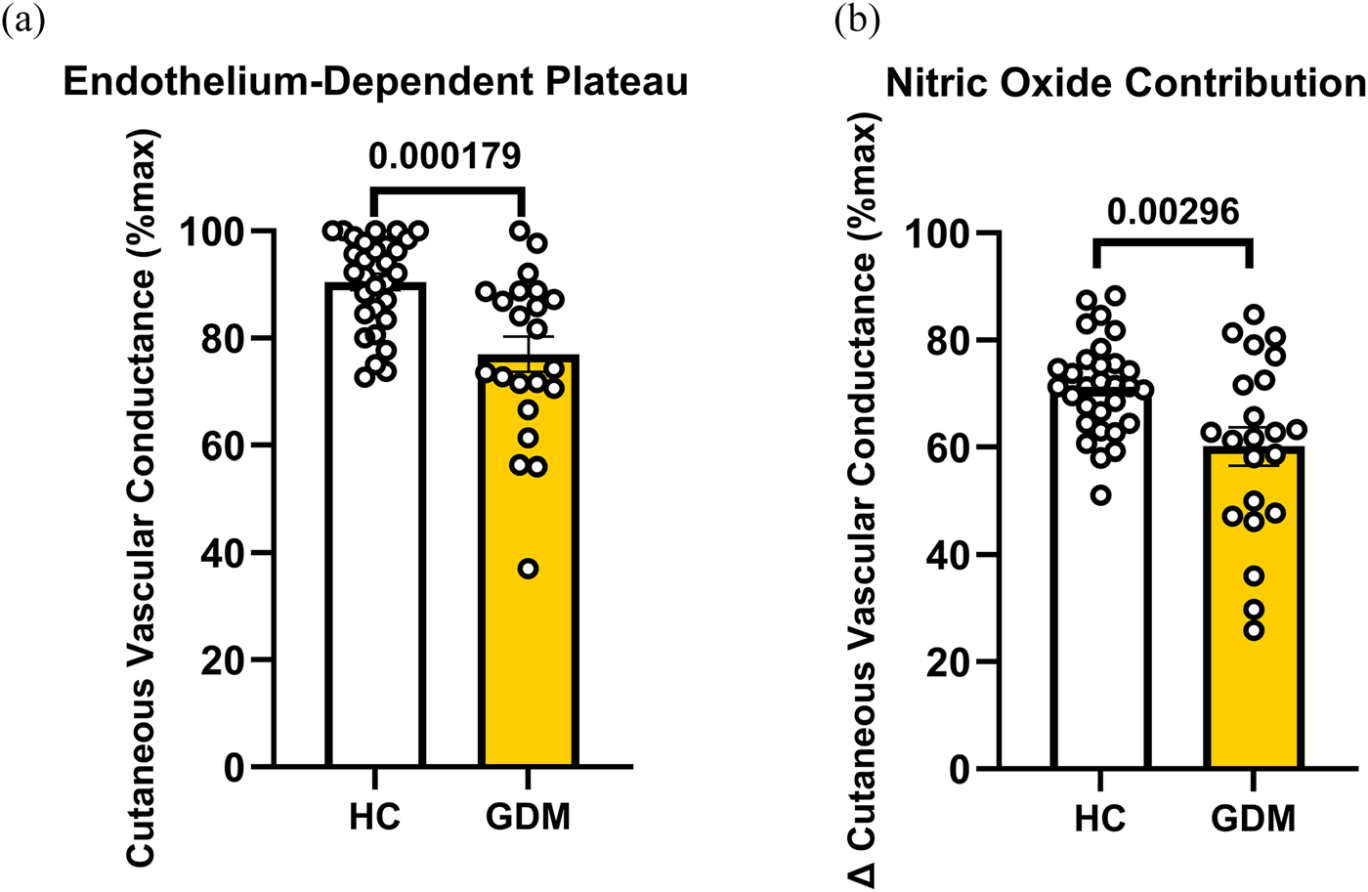
Endothelium‐dependent plateau (a) and nitric oxide contribution to the plateau (b). Mean ± SEM cutaneous vascular conductance (% of maximum) response to a standard local heating protocol in the cutaneous microvasculature (42°C), depicting the endothelium‐dependent plateau (*P *= 0.000179) and the nitric oxide contribution to the plateau (*P *= 0.00296) in women with a history of gestational diabetes mellitus (GDM, *n* = 22 participants) compared with healthy control (HC, *n* = 30 participants) women who had an uncomplicated pregnancy. Nitric oxide‐dependent dilatation was calculated by subtracting the *N* ^G^‐nitro‐l‐arginine methyl ester plateau from the endothelium‐dependent plateau (Δcutaneous vascular conductance, % of maximum).

Figure 3 presents the endothelium‐dependent plateau phase of the local heating response (Figure 3a) and the NO contribution to the plateau (Figure 3b). The endothelium‐dependent plateau (CVC, %max: HC, 90.5 ± 8.6 vs. GDM, 77 ± 15.3; *P *= 0.000179, ANCOVA *P *< 0.001) was attenuated in GDM. The NO contribution to the plateau (ΔCVC, %max: HC, 71.3 ± 8.8 vs. GDM, 60.2 ± 16.6; *P *= 0.00296, ANCOVA *P *< 0.001) was also attenuated in GDM.

## 4 DISCUSSION

The primary finding of this study was that otherwise healthy women with a history of GDM demonstrated no differences in local heating‐induced sensory nerve‐mediated microvascular vasodilatation compared with women who had an uncomplicated pregnancy, despite reductions in endothelium‐ and NO‐dependent dilatation. Using the human cutaneous circulation as an in vivo model to examine peripheral microvascular function (Debbabi et al., 2010; Holowatz et al., 2008; Kenney et al., 2013), we provide the first direct evidence that cutaneous sensory nerve‐mediated vasodilator function is preserved in otherwise healthy women with a history of GDM compared with HC women.

Gestational diabetes is characterized by glucose intolerance during pregnancy, and despite the return to normal clinical values for fasting glucose and HbA1c after pregnancy, women with a history of GDM are seven times more likely to develop T2DM compared with women who had an uncomplicated pregnancy (Bellamy et al., 2009). T2DM is associated with microvascular endothelial dysfunction and sensory nerve dysfunction (Feener & King, 2001; Muris et al., 2012; Nacci et al., 2009; Pek et al., 2017; Williams et al., 1996). In fact, the most frequent complication of T2DM is diabetic peripheral neuropathy (Røikjer & Ejskjaer, 2022). We have demonstrated that despite the return to normal clinical values for fasting glucose and HbA1c, healthy women with a history of GDM have reduced endothelial function in the cutaneous microvasculature (Schwartz et al., 2024; Stanhewicz et al., 2022). However, to date, it is unknown whether sensory nerve‐mediated vascular responses are reduced similarly in these women. To investigate sensory nerve‐mediated vasodilatory function in otherwise healthy women with a history of GDM, we used the cutaneous microvascular response to local heating. Local heating of the skin activates two independent vasodilatory mechanisms in the cutaneous microvasculature: a fast‐responding sensory nerve‐mediated peak and a slower‐responding sustained plateau that relies on a functional endothelium. The initial rise in skin blood flow is transient, is mediated predominantly through local, thermally sensitive afferents acting in an axon reflex independent of the CNS (Minson et al., 2001; Stanhewicz et al., 2022) and is largely NO independent (Kellogg et al., 1999; Minson et al., 2001; Sokolnicki et al., 2007, 2009). Because we previously identified reductions in endothelium‐ and NO‐dependent dilatation responses to local heating in women with a history of GDM, we sought to examine whether cutaneous sensory nerve‐mediated responses were reduced similarly in response to local heating in the present study. Contrary to our hypothesis, women with a history of GDM had a preserved initial sensory nerve‐mediated peak blood flow response to local heating, in both absolute and normalized CVC in the cutaneous microvasculature. These findings suggest that otherwise healthy women with a history of GDM do not have decrements in sensory nerve‐mediated blood flow responses in the peripheral microvasculature.

The cutaneous local axon reflex is mediated primarily by TRPV‐1 channels on afferent nociceptive neurons (Wong & Fieger, 2010). TRPV‐1 channels mediate 50% of the sensory nerve‐mediated response to local heating (Carter & Hodges, 2011; Hodges et al., 2016; Minson et al., 2001) by inducing the release of calcitonin gene‐related peptide and substance P (Wallengren et al., 1987). Stimulating TRPV channels with agonists such as capsaicin (Charkoudian et al., 2001) or GSK101 (Fujii et al., 2019) causes sensory nerve‐mediated dilatation in the cutaneous microvasculature. However, specifically targeting TRPV channel activation or inhibition has not been explored in the context of GDM history. Downstream of TRPV channels, endothelium‐derived hyperpolarizing factors and NO contribute to the local axon reflex (Brunt & Minson, 2012; Minson et al., 2001). Participants in the present study demonstrated a reduction in the NO‐dependent contribution to the endothelium‐dependent plateau response. However, we did not directly examine endothelium‐derived hyperpolarizing factor‐dependent dilatation or the NO contribution to the axon reflex in the present study. Although our data suggest that a history of GDM does not alter the magnitude of the local axon reflex, we did not delineate the mechanisms driving this response, and it is possible that alterations in upstream mechanisms mediating the axon reflex response are altered in women with a history of GDM compared with HC women.

We enrolled HC and GDM women who were within 5 years of pregnancy and had clinically normal fasting plasma glucose and HbA1c values at the time of the study. Because we previously observed reduced endothelium‐dependent dilatation responses in healthy women with a history of GDM, we hypothesised that sensory nerve‐mediated microvascular dysfunction could underlie future overt T2DM and potential subsequent diabetic peripheral neuropathy in this population of women. Chronic hyperglycaemia is considered a main mechanism driving the development of peripheral neuropathy in diabetes (Yagihashi et al., 2011). However, it is unclear whether short‐term GDM‐induced hyperglycaemia impairs sensory nerve function in women with a history of GDM, independent of T2DM. By design, our cohort of women did not have hyperglycaemia at the time of testing and experienced short‐term hyperglycaemia only during the final 12–16 weeks of pregnancy. The presence of normal glucose tolerance at the time of testing may partly explain why our cohort of women with a history of GDM did not exhibit dysfunction of peripheral nerves. It is unclear whether sensory nerve dysfunction might develop later during the postpartum period (perhaps >5 years after delivery), independent of T2DM development.

Evidence suggests that impairments in sensory nerve function (i.e., reduced plantar sensation) exist during pregnancy complicated by GDM (Doğan & Demir Çaltekin, 2023). However, it is unknown whether acute hyperglycaemia (12–16 weeks) seen in GDM induces long‐term impairments in sensory nerve function. Chronic hyperglycaemia contributes to the development of diabetic peripheral neuropathy within the first 8–10 years of T2DM (Bodman et al., 2026), and its rate of progression can vary depending on glycaemic management strategies that are adopted (Yagihashi et al., 2011). There is no clearly defined clinical duration of chronic hyperglycaemia that is known to elicit peripheral neuropathy in the context of diabetes. However, the clinical manifestations of peripheral neuropathy can appear within 3–5 years (Allen et al., 1997) of sustained high blood sugar, suggesting that the time frame by which chronically high glucose exposure damages nerves varies greatly. Importantly, restoration of glycaemic control fails to reverse peripheral neuropathy in diabetic patients (Ang et al., 2014; Yang et al., 2025). It is plausible that the ∼12–16 weeks of hyperglycaemia during GDM is not enough time to induce pathophysiological changes in sensory nerves that can be detected after clinical symptoms of hyperglycaemia have resolved. However, this short‐term exposure of hyperglycaemia during GDM might initiate pathways (e.g. advanced glycation end‐products, inflammation and oxidative stress; Strand et al., 2024) that might eventually elicit detectable impairments in sensory nerve function later in life. Future work examining sensory nerve function >5 years postpartum but before development of T2DM or CVD could provide insight into the timing of disease progression. However, our data suggest that a history of GDM does not impair sensory nerve‐mediated peripheral microvascular function ≤5 years postpartum and prior to the development of overt T2DM.

Compiling our data from previous studies (previously published data from *n* = 20 HC participants and *n* = 7 GDM participants) with additional studies conducted in our laboratory to achieve a total sample size of 30 HC and 22 GDM participants, we found that women with a history of GDM exhibit reduced microvascular endothelial function, as evidenced by a reduced endothelium‐dependent plateau response to local heating, mediated by reductions in NO‐dependent dilatation. In contrast to the initial sensory nerve‐mediated peak, the secondary endothelium‐dependent plateau is primarily NO dependent (60%–80%) and does not rely on the axon reflex for full expression. This distinction in mechanisms involved in the cutaneous local heating response is well demonstrated by the fact the women with a history of GDM exhibited differential responses in each phase of the local heating response in the present study: preserved cutaneous sensory nerve‐mediated function paired with reduced endothelium‐ and NO‐dependent dilatation. Our results suggest that the mechanisms that reduce endothelium‐ and NO‐dependent dilatation do not also reduce sensory nerve‐mediated dilatation in the peripheral microvasculature of women with a history of GDM.

In the present study, we found that the GDM group had significantly higher maximal CVC compared with the HC group. Higher absolute maximal CVC could potentially indicate that the GDM group had greater smooth muscle sensitivity to NO (microdialysis perfusion of sodium nitroprusside) compared with the HC group or that the women with a history of GDM had greater cutaneous microvessel density in the areas we assessed. Normalization to maximal CVC is standard practice with intradermal microdialysis and accounts for the heterogeneity of vessel density across the cutaneous vascular beds. In our previous studies, we have found reductions in endothelium‐ and NO‐dependent dilatation without group differences in maximal CVC when comparing women with a history of GDM against women with a history of uncomplicated pregnancy using this approach (Maurer et al., 2026; Schwartz et al., 2024; Stanhewicz et al., 2022). Our normalized data in the present manuscript agree with these previous findings. However, in the present study, we did not find group differences in the absolute CVC response to local heating, which suggests that the absolute conductance response to local heating is the same in these cohorts, whereas the response relative to site‐specific maximal potential is impaired. Importantly for the present analysis, we did not find group differences in the initial peak response to local heat in either absolute CVC or CVC normalized to maximum, supporting our primary finding that sensory nerve‐mediated dilatation is not different between women with a history of GDM and women with a history of uncomplicated pregnancy.

### 4.1 Limitations

We assessed cutaneous sensory nerve‐mediated microvascular function indirectly, by quantifying the initial sensory nerve‐mediated peak CVC response to non‐painful local heating of the skin. We did not directly assess the sensory nerve contribution to this vasodilatory response by blocking local cutaneous nerves with topical anaesthetic, such as an eutectic mixture of local anaesthetic cream (Minson et al., 2001) or lignocaine (Strom et al., 2010). Although our data suggest that sensory nerve‐mediated responses in women with a history of GDM are preserved, future studies using methods to examine sensory nerve function mechanistically might be warranted in this population.

Our cohort of GDM women exhibited higher fasting glucose, HOMA‐IR and HbA1c compared with the HC women. However, fasting insulin and HOMA‐IR were assessed in only a subset of participants. By design, the plasma glucose concentrations and HbA1c values in both the HC and GDM groups were all within clinically normal ranges, and the presence of glucose intolerance (HbA1c > 5.7% and/or fasting glucose > 100 mg dL^−1^), CVD and metabolic disease were exclusion criteria in both groups (Bennett et al., 2007). We have previously identified that differences in glucose, HOMA‐IR and HbA1c do not explain the differences in our in vivo measures of microvascular function (Maurer et al., 2026; Schwartz et al., 2024; Stanhewicz et al., 2022). Importantly, inclusion of fasting glucose and HbA1c as covariates in our statistical approach did not alter our conclusions, suggesting that these differences in markers of glucose control were not driving our primary findings.

Our cohort of GDM women also exhibited higher BMI than the HC women. The average BMI of the HC group was in the overweight category (25–30 kg m^−2^), whereas the average BMI of the GDM group was in the class 1 obesity category (30–35 kg m^−2^). However, both groups included women with BMI in the overweight and obese categories. It is plausible that higher BMI might contribute to reductions in microvascular function and differentially affect sensory nerve‐mediated function (Buschbacher, 1998; Sand et al., 2025), independent of GDM history; and obesity‐related neuropathy has been demonstrated regardless of glycaemic status (Elafros et al., 2024) in humans. To this end, we did find that BMI was a significant covariate for sensory nerve‐mediated peak and endothelium‐dependent plateau responses across groups. However, even when adjusting for BMI, we did not detect significant differences in sensory nerve‐mediated function between our groups, and differences in endothelial function remained significant. These data are consistent with our previous finding that group differences in BMI do not explain differences in our measures of microvascular function (Maurer et al., 2026). Future work should examine the role of obesity in moderating microvascular function in women. Our analysis suggests that our primary findings in the present study are not driven by group differences in BMI.

Although a history of GDM is associated with lipid abnormalities (Wang et al., 2023), and hypertriglyceridaemia has been linked to sensory neuropathy (Iqbal et al., 2025), our cohort of women exhibited normal triglyceride levels (<150 mg dL^−1^; National Heart, Lung, and Blood Institute, 2023). However, it is important to acknowledge that triglyceride levels within the ‘normal’ range have been linked to an increased risk of T2DM (Szili‐Torok et al., 2022). Although we did not control for menstrual cycle phase or contraceptive use in the present study, previous work demonstrates that menstrual cycle phase does not influence microvascular responses assessed in the cutaneous vascular bed (D'Agata et al., 2021; Ketel et al., 2009; Rossi et al., 2009).

## 5 CONCLUSION

We found that the cutaneous sensory nerve‐mediated response to local heating was preserved in women with a history of GDM compared with HC women, suggesting that the mechanisms reducing endothelium‐ and NO‐dependent dilatation after GDM do not also reduce sensory nerve‐mediated dilatation in these women. We investigated this high‐risk cohort of women specifically after pregnancy, when their glucose tolerance had been restored to normal and before development of T2DM or CVD. Further work investigating alternative mechanisms of microvascular dysfunction and sensory nerve‐mediated function in this high‐risk cohort are warranted.

## AUTHOR CONTRIBUTIONS

Research design: Grace S. Maurer, Anna E. Stanhewicz, Diana I. Jalal and Kelsey S. Schwartz. Data analysis and interpretation: Grace S. Maurer. Manuscript preparation: Grace S. Maurer, Kelsey S. Schwartz, Diana I. Jalal and Anna E. Stanhewicz. Funding and research design: Anna E. Stanhewicz and Grace S. Maurer. Data interpretation: Grace S. Maurer, Diana I. Jalal and Anna E. Stanhewicz. Data collection, analysis and interpretation: Grace S. Maurer and Kelsey S. Schwartz. All authors have read and approved the final version of this manuscript and agree to be accountable for all aspects of the work in ensuring that questions related to the accuracy or integrity of any part of the work are appropriately investigated and resolved. All persons designated as authors qualify for authorship, and all those who qualify for authorship are listed.

## CONFLICT OF INTEREST

The authors have no conflicts of interest to disclose.

## GENERATIVE AI STATEMENT

No generative AI tools were used in the preparation of this manuscript.

## Data Availability

Data supporting these findings are available from the corresponding author upon reasonable request and under a Data Use Agreement with the University of Iowa.
